# GC-MS Evaluation, Antioxidant Content, and Cytotoxic Activity of Propolis Extract from Peninsular Malaysian Stingless Bees, *Tetrigona Apicalis*

**DOI:** 10.1155/2020/8895262

**Published:** 2020-12-09

**Authors:** Wan Ahmad Syazani Mohamed, Noor Zafirah Ismail, Eshaifol Azam Omar, Nozlena Abdul Samad, Siti Khadijah Adam, Sharlina Mohamad

**Affiliations:** ^1^Integrative Medicine Cluster, Advanced Medical and Dental Institute, Universiti Sains Malaysia, SAINS@BERTAM, 13200 Kepala Batas, Penang, Malaysia; ^2^Pharmacology Unit, Department of Human Anatomy, Faculty of Medicine and Health Sciences, Universiti Putra Malaysia, 43400 UPM Serdang, Selangor, Malaysia

## Abstract

**Introduction:**

Propolis has been used traditionally in several countries for treating various diseases as it possessed healing properties including antioxidant and anticancer qualities. In Peninsular Malaysia, *Tetrigona apicalis* is one of the species of stingless bees mainly found in virgin jungle reserves which largely contribute to propolis production. Therefore, this study is designed to evaluate the phytochemical contents, antioxidant properties, and the cytotoxic effect of ethanolic crude of propolis extract against MCF7 and MCF 10A cell lines.

**Method:**

The ethanolic extract of propolis (EEP) was extracted using 80% ethanol. Identification of phytochemical contents and antioxidant properties of EEP was analysed by gas chromatography-mass spectrometry (GC-MS) and using 2, 2′-azinobis (3-ethylbenzothiazoline-6-sulphonic acid) (ABTS) method, respectively. The EEP cytotoxic activity was evaluated on MCF7 and MCF 10A using 3-(4, 5-dimethylthiazol-2-yl)-2, 5-diphenyltetrazolium bromide (MTT) assay.

**Results:**

Phytochemical contents of EEP demonstrated 28 compounds in which caryophyllene (99%), *β*-amyrin (96%), *α*-amyrin (93%), and caryophyllene oxide (93%) were the main compounds. The percentage of ABTS^+^ scavenging activity of EEP showed an inhibition of 9.5% with half-inhibitory concentration (IC_50_) value of 1.68 mg/mL. The EEP reduced MCF7 cells viability at IC_50_ value of 62.24 *μ*g/mL, 44.15 *μ*g/mL, and 32.70 *μ*g/mL at 24, 48, and 72 hours, respectively. The IC_50_ value of MCF 10A was 49.55 *μ*g/mL, 56.05 *μ*g/mL, and 72.10 *μ*g/mL at 24, 48, and 72 hours, respectively. The EEP cytotoxic effect of *T. apicalis* was more selective towards MCF7 at 72-hour incubation with a selectivity index (SI) of 2.20.

**Conclusion:**

The EEP has been shown to have antioxidants and potential bioactive compounds and inhibited proliferation of the MCF7 cells. Further studies on the EEP role in the apoptosis pathway and its screening towards other cell lines will be evaluated.

## 1. Introduction

Propolis is popularly known as one of the traditional herbal medicines used worldwide. Propolis is composed of a collection of sticky resinous materials from different plant sources that function as a sealant for hole or cracks in the beehive. The word propolis comes from the Greek word “pro” meaning barrier or in defence and “polis” meaning city, or in full “defence of the city (or the hive)” [1]. Propolis has been effectively used in folk medicine since ancient times to treat cold sores and abscesses. Fortified with its unique pharmacological properties, several studies such as anti-inflammatory, antioxidant, antifungal, antibacterial, antihepatotoxic, and anticancer have been done and successfully published [1–3].

Research on propolis has become the topic of interest due to its bioactive compounds and vital biological activities, particularly in Southeast Asian countries [4]. In Malaysia, multiple researches have been conducted to observe the effectiveness of propolis for some biological activities. The detection of phytochemical compounds and antioxidant activity of Malaysian propolis was successfully discussed in several studies using gas chromatography-mass spectrometry (GC-MS) and thin-layer chromatography (TLC) [5–9].

The abundance of stingless bee species in Malaysia, 17 to 32 of them well-known [10], has led to many opportunities to research each type of propolis, based on its precise species. This study focuses on *Tetrigona apicalis. Tetrigona apicalis* first mentioned by Wallace and Smith [11] can be found mainly in the subtropical areas of Southeast Asia and Indo-Malaya/Australasia [12]. *T. apicalis* was selected for this study, as this particular species can easily be found in virgin jungle reserves, especially in Peninsular Malaysia [13]. Unlike other main species of Malaysian stingless bees that are normally kept for meliponiculture, such as *Heterotrigona itama* and *Geniotrigona thoracica* [10], *T. apicalis* is native to the wild and known for its efficacy as a potent pollinator group in most ecosystems [14].

Rosli et al. [8], Gapar [15], and Asem et al. [16] have investigated the antioxidant activity of propolis extract from *T. apicalis* via 2, 2-diphenyl-1-picrylhydrazyl (DPPH) and 2, 2′-azinobis (3-ethylbenzothiazoline-6-sulphonic acid) (ABTS^+^) assay. Gapar [15] also correlated the antioxidant activity of *T. apicalis* propolis extract with total phenolic and flavonoid content.

The propolis extract of *T. apicalis* has also been well investigated in terms of its cytotoxic activities. Mat Nafi [9] found that *T. apicalis* did not exhibit any cytotoxic activity against HeLa (cervical cancer cells), MDA-MB-231 (breast cancer cells), and SK-UT-1 (uterine leiomyosarcoma cells) cancer cells. In addition, Gapar [15] also found that propolis extract of *T. apicalis* inhibited almost 50% of HeLa cells through apoptosis induction.

To date, there exists no study on the cytotoxicity of *T. apicalis* on MCF 7 (breast cancer cells lines), a hormone-dependent cell, and MCF 10A, nontumour human mammary epithelial cell lines. The *in vitro* cytotoxic assays for both cell types are crucial to determine whether the *T. apicalis* propolis extract is capable of working as a potential anticancer agent and in turn reducing toxicity towards noncancerous cells [17]. At the same time, studies specifically focused on the phytochemical screening of *T. apicalis* propolis extract for the identification of bioactive compounds for antioxidant and anticancer properties are lacking. Thus, this study aims to determine the phytochemical compounds of *T. apicalis* propolis extract along with ABTS^+^ radical scavenging activity and cytotoxic effect against MCF7 and MCF 10A.

## 2. Methodology

### 2.1. Materials

Dulbecco's Modified Eagle Medium (DMEM), Roswell Park Memorial Institute (RPMI-1640), fetal bovine serum (FBS), trypsin/EDTA, penicillin-streptomycin, horse serum, hydrocortisone, Epidermal Growth Factor (EGF), insulin, phosphate buffer saline (PBS), and 3-(4, 5-dimethylthiazol-2-yl)-2, 5-diphenyltetrazolium bromide (MTT) were purchased from Gibco-BRL. ABTS^+^ aqueous solution, potassium persulfate, ethanol, Trolox, methanol, and dimethyl sulfoxide (DMSO) were obtained from Sigma Aldrich (St. Louis, USA).

### 2.2. Sample Collection and Identification of *T. apicalis*

Propolis from the *T. apicalis* was collected at Tanjung Malim, Perak, Malaysia, by collecting the bee nest's inner part as described by Bonamigo et al. [18] with some modifications. The sample was kept in a plastic container and labelled. In order to identify the species of the stingless bees, the bee samples from the hive were taken as well. Alcohol swabs (soaked with 70% isopropyl alcohol) with scanty drops of 5% glacial acetic acid were prepared and placed in a killing jar prior to bee collection. The bee sample was put into the killing jar and the cover was closed tightly. The dead bees were put into the specimen container containing silica gel for further identification. Identification of the sample was completed by Centre for Insect Systematics (CIS), School of Environmental and Natural Resource Sciences, Faculty of Science and Technology of Universiti Kebangsaan Malaysia (UKM).

### 2.3. Preparation of Propolis Extract

Raw propolis samples with dust and the dead bees were removed physically from the samples. The preparations of the sample were in accordance with the method by Kothai and Jayanthi [19] with minor modifications. About 10 g powdered sample of *T. apicalis* propolis was extracted using 80% ethanol and stirred continuously at 400 rpm for 24 hours. The suspensions of the samples were subjected to centrifugation at 3000 rpm for 10 minutes. The extract was filtered using filter paper and concentrated using a rotary evaporator. The extracts were stored in a −20°C freezer. Thereafter, the extract was freeze-dried and reduced to powder form.

### 2.4. Gas Chromatography-Mass Spectrometry (GC-MS)

Agilent Technologies 6890N Network GC system was used for GC-MS evaluation of phytochemical contents of the *T. apicalis* propolis extract. Approximately, 1 mg of the extract was dissolved in 1 mL methanol before filtering with a 0.45 *μ*m Whatman nylon syringe filter. The extract was injected automatically in a splitless mode. The starter temperature was placed at 70°C and kept for 2 minutes. The temperature was further increased to 160°C (with a rate of 10°C/min) and maintained for 5 minutes. Eventually, the temperature was escalated to 270°C (with a rate of 20°C/min) and stabilised for 8 minutes. The compounds were selected based on the comparison from the National Institute of Standards and Technology (NIST) library. The compounds that showed 80% similarity with chemical compounds from NIST were selected for this study.

### 2.5. ABTS^+^ Radical Scavenging Assay

Determination of free radical scavenging activity in the ethanolic extract of propolis (EEP) was conducted by the method as described by Ismail et al. [6], Campos et al. [20], and Vongsak et al. [21] with minor modifications. Initially, 7 mM aqueous solution of ABTS^+^ and 2.45 mM potassium persulfate in water was prepared and reacted. The mixture was kept in the dark at room temperature for 12 to 16 hours. ABTS^+^ radical solution was diluted by reacting 1 mL ABTS^+^ radical with 50 mL ethanol to achieve the absorbance of 0.70 (±0.02) at 734 nm using a spectrophotometer (Biomate spectrophotometer, Thermo Fisher Scientific, USA).

Samples at concentrations ranging from 0.01 to 0.313 mg/mL were used. About 125 *μ*L of ABTS^+^ radical was mixed with 1.25 *μ*L of samples in a 96-well plate. The mixture was then incubated in the dark for 6 minutes at 37°C. All sample concentrations were tested in triplicates. The percentages of scavenging effects were measured by the equation as follows [22]:

(1) $$\begin{array}{c} \text{Inhibition }\left(\%\right)=\frac{\left(A_{1}-A_{2}\right)}{A_{1}}\times 100\%, \end{array}$$

where *A*_1_ is the absorbance of control and *A*_2_ is the absorbance of samples. Each concentration was done in triplicate, and the mean half-maximal inhibitory concentration (IC_50_) value was counted as mean ± standard deviation (SD). The positive control (Trolox) was treated under the same conditions as the samples.

### 2.6. Cytotoxic Assay

#### 2.6.1. Cell Culture Maintenance

The MCF7 cell line was cultured in complete RPMI-1640 medium (included with penicillin-streptomycin and FBS); meanwhile, the MCF 10A cell line was cultured in complete DMEM (horse serum, hydrocortisone, EGF, insulin, and PBS). Both cells were incubated in a 5% CO_2_ at 37°C for 48 to 72 hours until 80% confluency.

#### 2.6.2. MTT Assay

MTT assay was used to determine the cytotoxic effect of the extract using the method described by Aziz et al. [23]. Both cell lines were seeded at a density of 1 × 10^4^ cells/mL in 96-well plates. Both cells were incubated for 24 hours to allow for cell attachment and were treated with 100 *μ*L of extracts. The extracts were freshly prepared beforehand by diluting 100 mg of EEP in 1 mL of 100% DMSO. An amount of less than 0.5% of DMSO was used in this step to prevent any toxic effect on the cell for any insoluble extracts [24]. Extracts with eight different concentrations ranging from 0.975 to 125 *μ*g/mL, positive and negative controls, were prepared with three replicates to ensure the validity of the results. Tamoxifen was used as a positive control, while negative control used media alone with 0.5% DMSO.

Cytotoxicity extracts were recorded for each time point (24, 48, and 72 hours). After incubation, 10 *μ*L of MTT solution was added to each well plate and was incubated for another 4 hours to produce formazan. Each well was added with 100 *μ*L DMSO. The purple colour formed due to the dissolved formazan with DMSO corresponded to the number of viable cells [9]. The absorbance was measured at 570 nm with a spectrophotometer. The results were calculated as the mean values and SD in triplicate. The measurement of cell viability along with the IC_50_ was calculated using the formula as stated as follows [25]:

(2) $$\begin{array}{c} \text{cell viability }\left(\%\right)=\frac{\left[\text{average absorbance }\left(\text{sample}-\text{blank}\right)\right]}{\left[\text{average absorbance }\left(\text{negative control}-\text{blank}\right)\right]}\times 100. \end{array}$$

### 2.7. Selectivity Index

The selectivity index (SI) was calculated in order to determine the cytotoxic selectivity of the tested substances by conducting the equation, as mentioned as follows:

(3) $$\begin{array}{c} \text{SI}=\frac{{\text{IC}}_{50}^{\text{no cancer cells}}}{{\text{IC}}_{50}^{\text{cancer cells}}}, \end{array}$$

where SI > 2 was considered as high selectivity as suggested by Rashidi et al. [26].

### 2.8. Statistical Analysis

The statistical analysis was done systematically in three replicates, and these data were represented as mean values along with SD. Microsoft Excel was used to plot graphs (cell viability (%) versus concentration) in order to conclude the IC_50_ of the extract groups and positive control groups. A nonparametric (Kruskal Wallis) test was used to correlate the viability of the cell (%) between the treatments with the negative control. All variables were evaluated via SPSS and Microsoft Excel with *p* < 0.05 considered as significant.

## 3. Results

### 3.1. *T. apicalis* Propolis Extract

The percentage of yield of crude extract was measured with its physical appearance and recorded. The crude extract appeared whitish, and the samples were in powder form. The EEP sample from crude ethanolic extract yielded 57%.

### 3.2. Gas Chromatography-Mass Spectrometry

The results of GC-MS analysis are summarised in Table 1.

### 3.3. ABTS^+^ Radical Scavenging Activity

**Figure 1:**
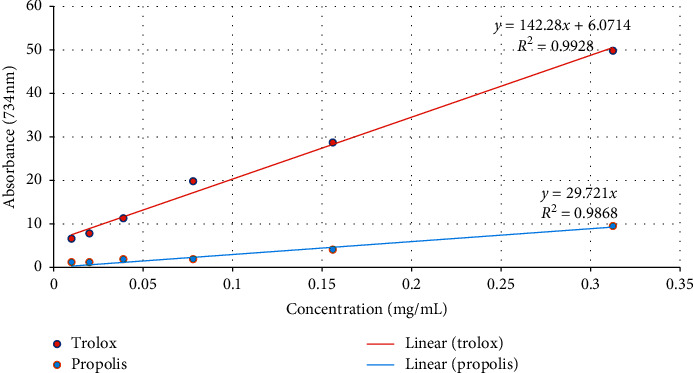
Linear graph showing the regression line of EEP and Trolox for ABTS+.

Figure 1 shows the different concentrations (0.01, 0.02, 0.039, 0.078, 0.156, and 0.313 mg/mL) of ABTS^+^ and control in the form of a linear regression graph. The IC_50_ value was determined by using a linear regression equation [27]. IC_50_ is described as the total antioxidant necessary to decrease the initial ABTS^+^ radical by half [28]. The IC_50_ of EEP and Trolox is further described in Table 2.

Based on Figure 1 and Table 2, the IC_50_ of EEP was 1.68 mg/mL with the maximal ABTS^+^ radical scavenging activity at 0.313 mg/mL and 9.5% inhibition corresponded to Trolox (49.8%).

### 3.4. Cytotoxicity of EEP

**Figure 2:**
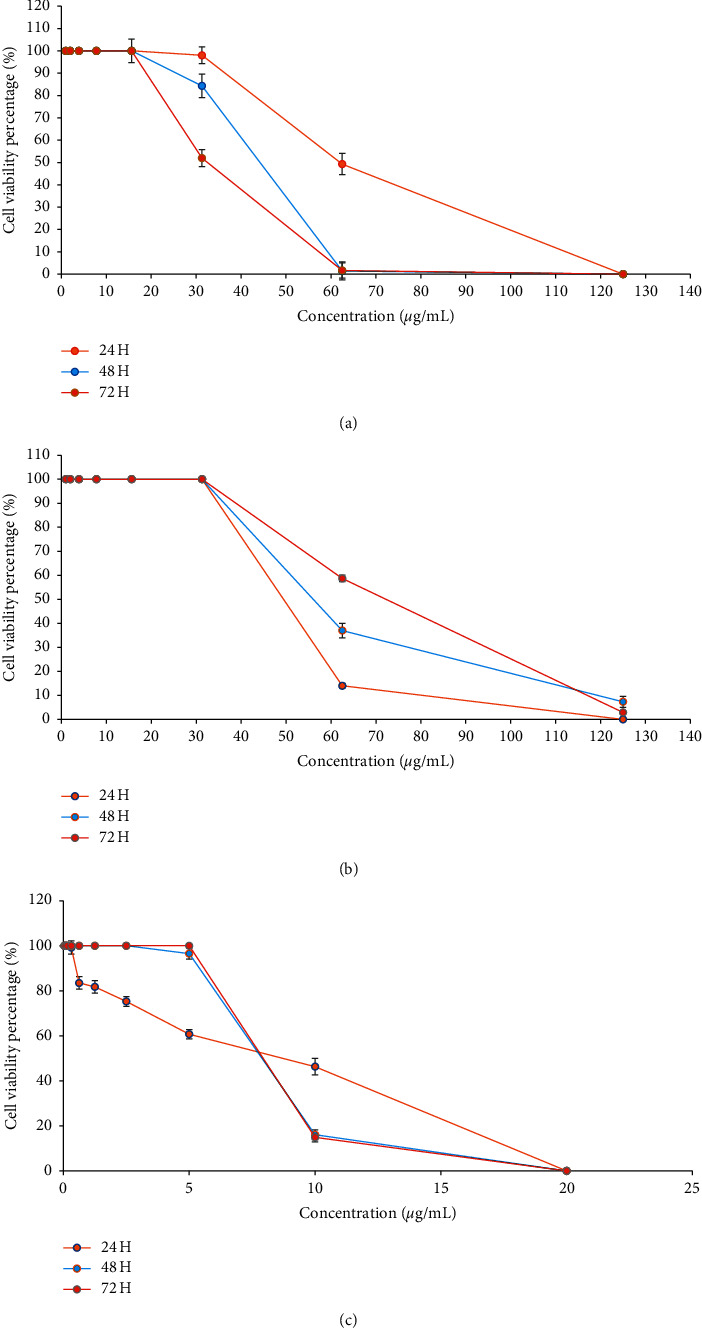
Graphs showing cell survival rates of (a) MCF7 cells and (b) MCF 10A cells with EEP. The cell survival rates with tamoxifen are shown in (c). Data presented as mean ± SD; ^*∗*^*p*<0.05 is considered a significant value.

Figures 2(a) and 2(b) show the different concentrations (0.975, 1.95, 3.9, 7.8, 15.6, 31.3, 62.5, and 125 *μ*g/mL) of EEP in MCF7 and MCF 10A, respectively.  Figure 2(c) represents the different concentrations (0.156, 0.313, 0.625, 1.25, 2.5, 5, 10, and 20 *μ*g/mL) of tamoxifen.

Based on Figure 2, treatment of both MCF7 and MCF 10A cells with different EEP concentrations resulted in a concentration-dependent effect. It can be observed that MCF7 cells that were treated with concentrations of 0.975, 1.95, 3.9, 7.8, and 15.6 *μ*g/mL showed 100% ± 0.02 cell survivals, for three independent time points. At 31.3 *μ*g/mL (with 98% ± 2.75, 84.2% ± 2.22, and 51.9% ± 2.87 for 24, 48, and 72 hours, resp.) and at 62.5 *μ*g/mL (for 24 hours with 49.3% ± 2.78), EEP started to inhibit proliferation of the cells and interrupted recovery. At higher concentrations (125 *μ*g/mL of 24 hours and 62.5 and 125 *μ*g/mL of 48 hours and 72 hours), it shows that the EEP completely inhibited proliferation and prevented recovery.

For MCF 10A cells, different EEP concentrations (0.975, 1.95, 3.9, 7.8, 15.6, and 31.3 *μ*g/mL) showed 100% ± 0.02 cell survival for 24, 48, and 72 hours, respectively. At 62.5 *μ*g/mL, all three time points demonstrated a decline in cell proliferation (with 14.1% ± 1.05, 37.0% ± 3.01, and 58.6% ± 1.35 for 24, 48, and 72 hours, resp.). At 125 *μ*g/mL, cell proliferation was completely inhibited at 24 hours and showed a cell survival rate of 7.3% ± 2.3 (for 48 hours) and 2.9% ± 0.58 (for 72 hours).

In terms of tamoxifen, the concentration of 0.156 *μ*g/mL showed a 100% ± 0.02 cell survival rate for 24, 48, and 72 hours, respectively. For 24 hours, the tamoxifen concentration of 0.313, 0.625, 1.25 2.5, 5, and 10 *μ*g/mL showed a gradual decline in cell proliferation (with 99.2% ± 2.91, 83.4% ± 2.74,81.7% ± 2.77,75.3% ± 2.18,60.7% ± 2.04, and 46.3% ± 3.61, resp.). Meanwhile, for 48 and 72 hours, the cell survival rate of 100% ± 0.02 remained steady across several concentrations (0.313, 0.625, 1.25, 2.5, and 5 (for 72 hours) *μ*g/mL). For 48 hours, the cell proliferation took place at a concentration of 5 *μ*g/mL (96.5% ± 2.45) and gradually continued at 10 *μ*g/mL (16.1% ± 2.13). For 72 hours, the declining cell proliferation was shown at 10 *μ*g/mL (14.9% ± 2.04). All three time points (24, 48, and 72 hours) showed complete inhibition of cell proliferation at a concentration of 20 *μ*g/mL.

The IC_50_ of the EEP on the MCF7 showed a cytotoxic level of 62.24 *μ*g/mL ± 0.016, 44.15 *μ*g/mL ± 0.02, and 32.70 *μ*g/mL ± 0.034 at 24, 48, and 72 hours, respectively, whereas the IC_50_ of the EEP on the MCF 10A showed a cytotoxic level of 49.55 *μ*g/mL ± 0.032, 56.05 *μ*g/mL ± 0.026, and 72.10 *μ*g/mL ± 0.027 at 24, 48, and 72 hours, respectively. The SI of MCF 10A/MCF7 is 0.8, 1.27, and 2.20 for all three time points of 24, 48, and 72 hours of treatment, respectively. The summary of IC_50_ and SI is shown in Table 3.

## 4. Discussion

**Figure 3:**
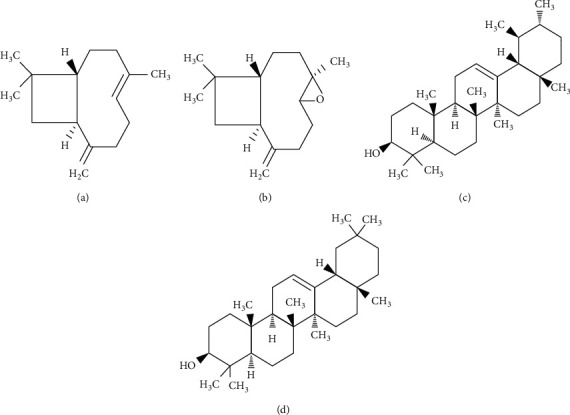
Chemical structure of sesquiterpene hydrocarbon, (a) *β*-caryophyllene [39], oxygenated sesquiterpenes, (b) *β*-caryophyllene oxide [40], and triterpenoids, (c) *α*-amyrin and (d) *β*-amyrin [41].

GC-MS analysis revealed 28 compounds from EEP derived from *T. apicalis* which were mainly predominant sesquiterpenes (C_15_H_24_). Major compounds of sesquiterpenes can be divided into two elements, sesquiterpene hydrocarbons, such as *β*-caryophyllene (caryophyllene) (99%), copaene (98%), and cyclohexane, 1-ethenyl-1-methyl-2,4-bis(1-methylethenyl)-, [1S-(1.alpha, 2.beta, 4.beta.)] (98%), and oxygenated sesquiterpenes, such as 1H-cycloprop[e]azulen-7-ol, decahydro-1 ,1, 7-trimethyl-4-methylene (98%) and *β*-caryophyllene oxide (caryophyllene oxide) (93%). These elements were reported in the previous literature for their high anti-inflammatory [29, 30], antioxidant [31, 32], antimicrobial [32], and anticancer activities [33, 34]. For instance, *β*-caryophyllene and *β*-caryophyllene oxide that constitute one of the major compounds in *T. apicalis* propolis extract act as potent anticancer and antioxidants as suggested by Fidyt et al. [35]. Apart from that, EEP of *T. apicalis* interestingly was found to have a high quality of triterpenoids such as *α*-amyrin (93%) and *β*-amyrin (96%) which were similar to the reports from Teixeira et al. [36]. Triterpenoids, typically *α*-amyrin and *β*-amyrin, were mentioned in several studies regarding their potential for anticancer properties as described by Barros et al. [37] and Mirunalini et al. [38]. The chemical structure of sesquiterpene hydrocarbon (*β*-caryophyllene), oxygenated sesquiterpenes (*β*-caryophyllene oxide), and triterpenoids (*α*-amyrin and *β*-amyrin) are shown in Figure 3. To the best of our knowledge, this is the first time these compounds are detected in Malaysian propolis from the *T. apicalis* species which is significantly important as these compounds may be potentially high in antioxidant and anticancer properties, which will be explained later. However, further evaluation of these phytochemical compounds might be needed with the use of appropriate standards such as using high-performance liquid chromatography (HPLC), liquid chromatography-mass spectrometry (LC-MS), and nuclear magnetic resonance (NMR) as it illustrates therapeutic significance.

GC-MS analysis in this study showed that the EEP of *T. apicalis* contains compounds with antioxidant and anticancer properties. Therefore, ABTS^+^ radical scavenging assay was selected to analyse the antioxidant activity of EEP since ABTS^+^ has a rapid kinetic reaction and intense response to antioxidants [42]. As suggested by Ibrahim et al. [5], the increment of antioxidant activity can be observed in the increase of radical scavenging activity that corresponded to a lesser value of IC_50_ of the extract. This is in agreement with our present study, where the EEP of *T. apicalis* possess inhibitory activity against ABTS^+^ radical with IC_50_ of 1.68 mg/mL although positive control (Trolox) was better with IC_50_ of 0.31 mg/mL. The graph in Figure 1 also showed that the radical scavenging activities were augmented, corresponding to the increase in the concentration of EEP. Previous studies by Rosli et al. [8] and Asem et al. [14] have also reported that *T. apicalis* propolis was very active in scavenging ABTS^+^ and DPPH radicals. These suggest that EEP has the potential to combat oxidative stress which is strongly attributed to the existence of crucial antioxidant compounds detected by the GC-MS analysis.

The previous cytotoxicity study on EEP of *T. apicalis* by Mat Nafi et al. [9] reported that EEP did not exhibit cytotoxic activity against different cancer cell lines. MDA-MB-231, SK-UT-1, and HeLa for 72 hours with IC_50_ of HeLa were 68 *μ*g/mL. Since EEP of *T. apicalis* consists of many bioactive compounds, a lower IC_50_ was predicted [23]. At 24 hours, EEP showed IC_50_ of 62.24 *μ*g/mL ± 0.016 and 49.55 *μ*g/mL ± 0.032 in MCF7 and MCF 10A cells, respectively. The IC_50_ was subsequently reduced at 48 and 72 hours in MCF7, whereas it was gradually increased in MCF 10A. The United States National Cancer Institute (USNCI) stated that crude extract with IC_50_ estimations less than 30 *μ*g/mL is a promising agent for the development of an anticancer medication, as mentioned by Quintans et al. [43]. Therefore, cytotoxicity of EEP in MCF7 at 72 hours had the most promising result for its potential as an anticancer agent since it has a low level of IC_50_ (32.70 *μ*g/mL ± 0.034). The target estimation of lesser IC_50_ (<30 *μ*g/mL) might be achieved if the time point exceeded 72 hours. These results indirectly reflect the relevance of EEP as a potential treatment in MCF7, also known to be ER, PR-positive, and HER2-negative breast cancer cell lines that are effective for hormonal treatment [44]. Based on the comparison with NIST, *β*-caryophyllene may play a role in inducing apoptosis in cancer cells with the catalytic activity involving DNA ladder and caspase-3. It has been known that *β*-caryophyllene augments the cytotoxicity of the isomers isocaryophyllene and *α*-humulene, particularly in MCF7 cancer cell lines [45, 46].

MCF 10A is originally nontumorigenic and thus SI was determined to find the best way to evaluate the selectiveness of treatment towards normal cell lines or cancer cell lines. As mentioned by Rashidi et al. [26], high selectivity against cancer cells was concluded where SI > 2, whereas if SI values fall in between 0 and 2, the treatment toxicity was otherwise not selective towards any cells. Based on our study, the SI at three time incubation points (24, 48, and 72 hours) was 0.8, 1.27, and 2.20, respectively. Therefore, it can be deduced that EEP showed general toxicity at 24–48 hours because the value is below 2.0 while it displayed selectivity when incubated further till 72 hours. As a result, EEP can be a potential candidate for a cytotoxic agent at 72 hours as evidenced by SI of 2.20. As MCF7 and MCF 10A were presented for the first time for the cytotoxic study of *T. apicalis*, both exhibited similar effectiveness of cytotoxic action which are time-dependent. The concentration-dependent EEP also plays a role in determining the effectiveness of EEP as discussed earlier. Therefore, these two key points are essential in determining the best potential of EEP to act as a cytotoxic agent towards MCF7.

## 5. Conclusion

EEP has been shown to have a high antioxidant level and potential bioactive compounds and can inhibit the proliferation of the MCF7 cells. The highest cytotoxic activities which corresponded to the lowest IC_50_ of MCF7 was obtained at 72 hours of EEP treatment with the best SI at the same time point. Further studies are recommended to determine the role of EEP in the apoptosis pathway and the effect of this extract on other cell lines.

## Figures and Tables

**Table 1:** Phytochemical compounds identified in *T. apicalis* propolis extract using GC-MS.

Peak	Compound	RT	MF	MW (g/mol)	Quality (%)
1	Undecane	5.602	C_11_H_24_	156.18	95
2	Cyclohexane, 1-ethenyl-1-methyl-2-(1-methylethenyl)-4-(1-methylethylidene)-	7.596	C_15_H_24_	204.35	90
3	alpha-Cubebene	7.68	C_15_H_24_	204.35	89
4	Copaene	7.895	C_15_H_24_	204.35	98
5	Cyclohexane, 1-ethenyl-1-methyl-2, 4-bis(1-methylethenyl)-, [1S-(1.alpha., 2.beta., 4.beta.)]-	7.979	C_15_H_24_	204.35	98
6	3H-3a,7-Methanoazulene, 2, 4, 5, 6, 7, 8-hexahydro-1, 4, 9, 9-tetramethyl-	8.111	C_15_H_24_	204.35	93
7	1H-Cycloprop[e]azulene, 1a, 2, 3, 4, 4a, 5, 6, 7b-octahydro-1, 1, 4, 7-tetramethyl-	8.145	C_15_H_24_	204.35	93
8	Caryophyllene	8.229	C_15_H_24_	204.35	99
9	alpha-Caryophyllene	8.451	C_15_H_24_	204.35	96
10	Naphthalene, 1, 2, 3, 4, 4a, 5, 6, 8a-octahydro-7-methyl-4-methylene-1-(1-methylethyl)-	8.562	C_15_H_24_	204.35	98
11	1, 6-Cyclodecadiene, 1-methyl-5-methylene-8-(1-methylethyl)-	8.625	C_15_H_24_	204.35	96
12	Bicyclogermacrene	8.722	C_15_H_24_	204.35	94
13	1, 3-Benzodioxole, 4-methoxy-6-(2-propenyl)-	8.806	C_11_H_12_O_3_	192.21	95
14	Naphthalene, 1, 2, 3, 5, 6, 8a-hexahydro-4,7-dimethyl-1-(1-methylethyl)-	8.84	C_15_H_24_	204.35	98
15	Naphthalene, decahydro-4a-methyl-1-methylene-7-(1-methylethenyl)-	9.09	C_15_H_24_	204.35	98
16	Tricyclo[6.3.0.0(2, 4)]undec-8-ene, 3, 3, 7, 11-tetramethyl-	9.188	C_15_H_24_	204.35	87
17	1H-Cycloprop[e]azulen-7-ol, decahydro-1, 1, 7-trimethyl-4-methylene-	9.236	C_15_H_24_O	220.35	99
18	Caryophyllene oxide	9.292	C_15_H_24_O	220.35	93
19	Cyclohexane, 1, 2-dimethyl-3, 5-bis(1-methylethenyl)-	9.375	C_14_H_24_	192.34	81
20	12-Oxabicyclo[9.1.0] dodeca-3,7-diene, 1, 5, 5, 8-tetramethyl	9.445	C_15_H_24_O	220.35	86
21	1H-Cycloprop[e]azulene, decahydro-1,1,7-trimethyl-4-methylene-	9.577	C_15_H_24_O	220.35	80
22	alpha-Cadinol	9.667	15_H_	222.37	93
23	Aristolene epoxide	10.98	C_15_H_24_O	220.35	83
24	1-Cyclohexene-1-butanal,.alpha.,2, 6, 6-tetramethyl-	11.571	C_14_H_24_O	208.34	90
25	1-Cyclohexene-1-butanal,.alpha.,2, 6, 6-tetramethyl-	11.634	C_14_H_24_O	208.34	93
26	4, 4, 6a, 6b, 8a, 11, 11, 14b-Octamethyl-1, 4, 4a, 5, 6, 6a, 6b, 7, 8, 8a, 9, 10, 11, 12, 12a, 14, 14a, 14b-octadecahydro-2H-picen-3-one	25.003	C_30_H_48_O	424.7	93
27	beta-Amyrin	25.621	C_30_H_50_O	426.7	96
28	alpha-Amyrin	26.886	C_30_H_50_O	426.7	93

Note: RT, retention time; MF, molecular formula; MW, molecular weight.

**Table 2:** The linear regression equation and IC50 value of EEP and Trolox.

Test solutions	Linear regression equation	IC_50_ value (mg/mL)
EEP	*y* = 29.721*x*	1.68
Trolox	*y* = 142.28*x* + 6.0714	0.31

**Table 3:** IC_50_ and SI at three time points (24, 48, and 72 hours) for MCF7, MCF 10A, and tamoxifen.

Incubation time (hours)	MCF7	MCF 10A	Tamoxifen	SI
24	62.24 *μ*g/mL ± 0.016	49.55 *μ*g/mL ± 0.032	9.05 *μ*g/mL ± 0.035	0.8
48	44.15 *μ*g/mL ± 0.02	56.05 *μ*g/mL ± 0.026	7.85 *μ*g/mL ± 0.02	1.27
72	32.70 *μ*g/mL ± 0.034	72.10 *μ*g/mL ± 0.027	7.85 *μ*g/mL ± 0.02	2.20

## Data Availability

The analysed data used to support the findings of this study are included within the article.

## Additional Points

*Key Points.* This journal article provides the effectiveness of *Tetrigona apicalis* propolis extract in promoting cytotoxic effect towards breast cancer cell line (MCF7) and nontumour human mammary epithelial cell lines (MCF 10A). The propolis extract of *T. apicalis* exhibited significant antioxidant properties and potential bioactive compounds and was capable of inhibiting the proliferation of the MCF7 cells. The highest cytotoxic activity of MCF7 was acquired at 72 hours of EEP treatment with the best SI at the same time point.
